# Prevalence of Group A *Streptococcus* in Primary Care Patients and the Utility of C-Reactive Protein and Clinical Scores for Its Identification in Thailand

**DOI:** 10.4269/ajtmh.19-0502

**Published:** 2019-12-30

**Authors:** Rachel Greer, Thomas Althaus, Clare Ling, Daranee Intralawan, Supalert Nedsuwan, Janjira Thaipadungpanit, Tri Wangrangsimakul, Christopher Butler, Nicolas Day, Yoel Lubell

**Affiliations:** 1 Mahidol-Oxford Tropical Medicine Research Unit, Faculty of Tropical Medicine, Mahidol University, Bangkok, Thailand;; 2 Nuffield Department of Medicine, Centre for Tropical Medicine and Global Health, University of Oxford, Oxford, United Kingdom;; 3 Shoklo Malaria Research Unit, Mahidol-Oxford Tropical Medicine Research Unit, Faculty of Tropical Medicine, Mahidol University, Mae Sot, Thailand;; 4 Social and Preventive Medicine Department, Chiang Rai Regional Hospital, Chiang Rai, Thailand;; 5 Department of Clinical Tropical Medicine, Faculty of Tropical Medicine, Mahidol University, Bangkok, Thailand;; 6 Clinical Trials Unit, Nuffield Department of Primary Care Health Sciences, University of Oxford, Oxford, United Kingdom

## Abstract

Pharyngitis is usually caused by a viral infection for which antibiotics are often unnecessarily prescribed, adding to the burden of antimicrobial resistance. Identifying who needs antibiotics is challenging; microbiological confirmation and clinical scores are used but have limitations. In a cross-sectional study nested within a randomized controlled trial, we estimated the prevalence and antibiotic susceptibility profiles of group A *Streptococcus* (GAS) in patients presenting to primary care with a sore throat and fever in northern Thailand. We then evaluated the use of C-reactive protein (CRP) and clinical scores (Centor and FeverPAIN) to identify the presence of GAS. One hundred sixty-nine patients were enrolled, of whom 35 (20.7%) had β-hemolytic *Streptococci* (BHS) isolated from throat swab culture, and 11 (6.5%) had GAS. All GAS isolates were sensitive to penicillin G. The median CRP of those without BHS isolation was 10 mg/L (interquartile range [IQR] ≤ 8–18), compared with 18 mg/L (IQR 9–71, *P* = 0.0302) for those with GAS and 14 mg/L (IQR ≤ 8–38, *P* = 0.0516) for those with any BHS isolated. However, there were no significant relationships between CRP > 8 mg/L (*P* = 0.112), Centor ≥ 3 (*P* = 0.212), and FeverPAIN ≥ 4 (*P* = 1.000), and the diagnosis of GAS compared with no BHS isolation. Identifying who requires antibiotics for pharyngitis remains challenging and necessitates further larger studies. C-reactive protein testing alone, although imperfect, can reduce prescribing compared with routine care. Targeted CRP testing through clinical scoring may be the most cost-effective approach to ruling out GAS infection.

## INTRODUCTION

Sore throat is a common presentation to primary care internationally, including Thailand. Approximately 40–80% of throat infections are caused by viruses, most commonly influenza, parainfluenza, coronavirus, rhinovirus, adenovirus, and enteroviruses, and antibiotics are ineffective for these infections. Bacterial pathogens are identified in 5–40% of cases, and group A *Streptococcus* (GAS) is the most common.^1,2^ A study of 290 Thai adults presenting to ambulatory care with acute upper respiratory tract infections (URTIs) found the presence of the following bacteria: 7.9% GAS, 9.2% non–group A β-hemolytic S*treptococcus* (BHS), 9.6% *Klebsiella pneumoniae*, 1.7% *Streptococcus pneumoniae*, 1.4% coagulase-negative *Staphylococcus*, 0.7% *Staphylococcus aureus*, 0.3% *Hemophilus influenzae*, 0.3% *Enterobacter* spp., and 0.3% *Proteus mirabilis*.^3^

In Chiang Rai, northern Thailand, pharyngitis or tonsillitis makes up over 30% of the respiratory tract infections presenting to government primary care units.^4^ National guidelines recommend the use of the Centor score to determine the need for antibiotics. When required, penicillin V and amoxicillin are recommended, or macrolides in the case of penicillin allergy.^5^ Antibiotics, typically amoxicillin, were prescribed to 88.4% of cases (14,621/16,539) from 2015 to 2016.^4^ Antibiotic resistance is an important problem in both the developed and developing world. In Thailand, an estimated 19,122 deaths were thought to be caused by multidrug-resistant hospital-acquired infections in 2010.^6^ Given that antibiotic use, even with narrow-spectrum agents, drives antimicrobial resistance (AMR), interventions that achieve safe reductions in antibiotic use are needed.

A 2016 Cochrane review compared antibiotic treatment against placebo for sore throats and found some reduction in symptom severity. However, average symptom duration was reduced by only 16 hours.^7^ Reducing the risk of complications is often used as a justification for antibiotic treatment, but a large cohort study from 2006 to 2009, albeit from a high-income setting (UK), found the risk of suppurative complications (otitis media, sinusitis, quinsy, and cellulitis) to be around 1% with or without antibiotic treatment. There were no cases of acute rheumatic fever or post-streptococcal glomerulonephritis.^8^ Nonetheless, these findings may not be applicable in low- and middle-income countries (LMICs) where prevalence of rheumatic heart disease is higher. The 2015 global burden of disease study found a high burden of rheumatic heart disease in Oceania, South Asia, and central sub-Saharan Africa. The burden is lower in Southeast Asia, and Thailand is considered non-endemic for rheumatic heart disease.^9^ Current guidelines recommend antibiotics for pharyngitis caused by GAS and that narrow-spectrum penicillins are most suitable.^10,11^ Concern is growing about increasing levels of GAS resistance to macrolide antibiotics.^12,13^

It is important to identify those most likely to benefit from antibiotic use and to reduce unnecessary antibiotic use to limit AMR; to achieve this, a variety of methods have been used, but with limited success. The best current reference standard for diagnosis of GAS is culture, although the length of time required to generate results limits its use to affect management decisions in primary care. Alternatives for use in primary care include rapid antigen testing, clinical scores such as Centor and FeverPAIN, and biomarkers of inflammation such as C-reactive protein (CRP). Studies assessing CRP’s performance in identifying the presence of GAS in patients presenting to primary care with sore throats or pharyngitis have produced mixed results, with some studies finding CRP predictive,^14–16^ whereas others have not.^2,17–20^ Rapid molecular tests for GAS are also being developed and have proved valuable in settings such as northern Australia, where a high burden of GAS, acute post-streptococcal glomerulonephritis, and acute rheumatic fever is evident.^21^

Our main objective was to evaluate the performance of CRP in identifying the presence of GAS among people consulting with sore throat and fever in Chiang Rai’s government-run primary care units. The secondary objectives were to estimate the prevalence of GAS and other key pathogens, study the antibiotic susceptibility of GAS isolates, and compare the performance of CRP against the Centor and FeverPAIN clinical scores.

## MATERIALS AND METHODS

This cross-sectional study of patients presenting with a sore throat was nested within a randomized controlled trial (RCT) evaluating the use of CRP to guide antibiotic prescription in those consulting with a fever (> 37.5°C) or history of fever.^22^ Patients were randomized to one of two CRP intervention groups (differing in the threshold used to recommend an antibiotic) or a control group. Patients were recruited from six government-run primary care units in Chiang Rai between June 2016 and August 2017. Chiang Rai is the most northern province in Thailand, bordering Laos PDR and Myanmar. It has a population of 1.3 million,^23^ of whom approximately 20% are from an ethnic minority group (Chiang Rai Highland People Development Center, personal communication). There are 32 government-run primary care units in Chiang Rai’s central district, serving a population of 240,000 in 2016.^23^ They are staffed primarily by registered nurses. The study sites are located within a 30-minute drive from the center of Chiang Rai.

The full list of the inclusion and exclusion criteria for the RCT is presented in the protocol summary on ClinicalTrials.gov (NCT02758821). In brief, eligible patients were aged 1 year or older presenting with a fever or history of fever and a total illness duration of less than 14 days. Patients were not excluded for prior antibiotic use. Recruitment was stratified by age (2 groups: children < 12 years adolescents and adults ≥ 12). Research nurses collected clinical data from the patients, including their presenting signs and symptoms and recent antibiotic use. Data on examination findings, diagnoses, and antibiotic prescription were collected from patients’ clinical records.

C-reactive protein was measured in the intervention group using NycoCard Reader II (Axis Shield, Norway; range for whole blood, 8–200 mg/L) on site using a finger-prick blood sample. The CRP results were available within minutes and fed back to the responsible clinicians. The CRP test for the control group was carried out later that day using venous blood samples, which were analyzed in the Chiang Rai Clinical Research Unit’s laboratory using the same CRP reader but without feedback to the responsible clinicians. All patients were followed up with a repeat point-of-care CRP test on day 5 and assessed for clinical recovery at days 5 and 14.

Patients with fever or a history of fever and sore throat were included in this nested study between November 2016 and August 2017 and had a throat swab taken. Centor^24^ (criteria = tonsillar exudate, tender anterior cervical lymphadenopathy or lymphadenitis, history of fever, and absence of cough) and FeverPAIN^25^ (criteria = fever in the last 24 hours, pus on the tonsils, attendance within 3 days of symptom onset, severely inflamed tonsils, and no cough or coryza) clinical scores were calculated retrospectively. Selection bias was reduced by recruiting consecutive eligible patients before any measurements.

Written informed consent or parental consent for those aged less than 18 years was obtained from all study participants, with additional assent obtained from those aged seven to less than 18 years in accordance with local guidelines. Ethical approval was obtained from the Oxford Tropical Research Ethics Committee, the Mahidol University Faculty of Tropical Medicine Ethics Committee, and Chiang Rai Provincial Public Health Office Research Ethics Committee.

### Laboratory.

Throat swabs were collected and placed in Amies transport medium with charcoal and stored at 0–4°C until processed at the Shoklo Malaria Research Unit (SMRU) laboratory, Tak Province, Thailand. Samples were plated onto blood agar and incubated at 35 ± 2°C in 5–10% CO_2_ for 20–24 hours. The presence of BHS was confirmed by Gram stain, catalase, and Lancefield grouping. GAS isolates were then tested for antimicrobial susceptibility by disk diffusion according to the Clinical and Laboratory Standards Institute criteria.^26^ The SMRU laboratory is not accredited but participates in external quality assurance (EQA) including the EQA program from the Department of Medical Sciences, Ministry of Public Health, Thailand, for bacterial identification and antimicrobial susceptibility testing.

Patients within the control group also had a nasopharyngeal swab taken, which was tested using TaqMan^®^ Array Card (TAC; Life Technologies^TM^, Waltham, MA).^27,28^ The assay was screened for 32 pathogens in a single run of multiplex reverse transcriptase real-time polymerase chain reaction (see Supplementary Material). This article fulfills the Microbiology Investigation Criteria for Reporting Objectively (MICRO) framework checklist requirements for standardized reporting of clinical microbiology data.^29^

### Statistical analysis.

Descriptive statistics were used to summarize demographic data using counts and percentages for categorical data and medians and IQR for continuous variables without a normal distribution. Ranksum was used to compare CRP values in those with positive throat swabs against those negative for BHS. Contingency tables were used to calculate the sensitivity and specificity of CRP to detect GAS against no BHS isolation, and Wilson’s method was used to calculate the 95% CIs for these estimates. Fisher’s exact test was used to estimate the correlation between CRP values > 8 mg/L, Centor scores ≥ 3, and FeverPAIN scores ≥ 4 and GAS detection compared with no BHS isolation because of low numbers per category. *P*-values < 0.05 were considered statistically significant. Analyses were conducted using STATA 15 (College Station, TX). Analyses were conducted on the full dataset without imputing missing data.

## RESULTS

A total of 1,182 patients with a documented fever or history of fever were recruited in Thailand into the main RCT between June 2016 and August 2017, and of 174 patients presenting with a sore throat between November 2016 and August 2017, 169 had a throat swab taken and were included in this nested study. Of these 169, 79 (46.8%) were male and 91 (53.9%) were aged less than 12 years. Antibiotic use before enrollment in the study was reported by 12/169 (7.1%, see Table 1). All patients completed follow-up at days 5 and 14.

**Table 1:** Patient characteristics and throat swab results

	All patients	BHS not isolated	Group A *Streptococcus*	Other BHS
Age adolescents and adults* (years), median (IQR); total = 78 (46.2%)	47 (20–59)	49 (24–60)	17.5 (12.5–45.5)	24 (20–59)
Age children (years), median (IQR); total = 91 (53.9%)	6 (4–9)	6 (4–9)	8 (7–9)	6 (4–7)
Male gender, *n*/*N* (%)	79/169 (46.8)	61/134 (45.5)	4/11 (36.4)	14/24 (58.3)
Antibiotic within last 2 weeks, *n*/*N* (%)	12/169 (7.1)	11/134 (8.2)	0/11	1/24 (4.2)
Chronic disease, *n*/*N* (%)	29/169 (17.2)	18/134 (13.4)	5/11 (45.5)	6/24 (25.0)
Duration of illness (days), median (IQR)	2 (2–4)	2 (2–4)	2 (2–4)	2 (2–3)
Throat examination, *n*/*N* (%)				
Abnormal	92/164 (56.1)	69/130 (53.1)	8/11 (72.7)	15/23 (65.2)
Exudate†	16/92 (17.4)	11/69 (15.9)	1/8 (12.5)	4/15 (26.7)
Injected or red†	68/92 (73.9)	52/69 (75.4)	5/8 (62.5)	11/15 (73.3)
Swollen or enlarged†	43/92 (46.7)	33/69 (47.8)	4/8 (50.0)	6/15 (40.0)

BHS = β-hemolytic *Streptococcus*.

* Defined as 12 years of age or older.

† Recorded as free text under details of abnormal examination, the denominator is those with an abnormal throat examination, and can have more than one.

In all, 164/169 (97.0%) patients were diagnosed with a single respiratory tract syndrome, of which 94/164 (57.3%) had a common cold, 42/164 (25.6%) had pharyngitis, 26/164 (15.9%) had tonsillitis, and 2/164 (1.2%) had bronchitis.

Antibiotics were prescribed to 53/169 (31.4%) patients at the index consultation. All but one antibiotic prescription was for amoxicillin (52/53, 98.1%), whereas one patient (1.9%) was prescribed roxithromycin. Antibiotics were prescribed to 2/94 (2.1%) of those with a clinical diagnosis of a common cold, 20/42 (47.6%) with pharyngitis, 25/26 (96.2%) with tonsillitis, and 2/2 (100%) with bronchitis.

### Swab results.

Beta-hemolytic *Streptococci* were isolated in 35/169 (20.7%) throat swabs: 11 (6.5%) GAS, four (2.4%) group B, four (2.4%) group C, one (0.6%) group F, 14 (8.3%) group G, and one (0.6%) non-groupable.

### Antibiotic susceptibility of GAS.

All GAS isolates were sensitive to ceftriaxone and penicillin G. There were resistance to erythromycin in 2/11 (18.2%), clindamycin in 2/11 (18.2%), and chloramphenicol in 2/11 (18.2%) isolates. A single isolate (9.1%) was resistant to all three antibiotics. Intermediate sensitivity to erythromycin was found in 1/11 (9.1%) and to chloramphenicol in a different 1/11 (9.1%) isolate (see Table 2).

**Table 2:** Antibiotic susceptibility of GAS

GAS isolate	Ceftriaxone	Chloramphenicol	Clindamycin	Erythromycin	Penicillin G
1	S	I	R	R	S
2	S	R	S	S	S
3	S	S	S	S	S
4	S	S	S	S	S
5	S	S	S	S	S
6	S	S	S	I	S
7	S	S	S	S	S
8	S	R	R	R	S
9	S	S	S	S	S
10	S	S	S	S	S
11	S	S	S	S	S

GAS = group A *Streptococcus*; I = intermediate; R = resistant; S = sensitive.

Clinical features of the patients with GAS are shown in Table 3. Of 11 patients with GAS, eight (72.7%) had abnormal throat examinations, four (36.4%) had pharyngitis, four (36.4%) had common colds, and three (27.3%) had tonsillitis.

**Table 3:** Individual clinical features, diagnoses, and management of patients with GAS-positive throat swabs

Patient	Age (years)	Abnormal throat examination	Clinical diagnosis	Other microbiological diagnosis from nasopharyngeal swab	CRP (mg/L)	Centor score*	FeverPAIN score	CRP group	Antibiotic prescription at index consultation	Antibiotic taken at days 0–14†	Symptoms resolved by day 14‡
1	13	Yes	Pharyngitis	N/A	12	1	3	Intervention	No	No	Yes
2	9	No	Common cold	GAS, *Moraxella catarrhalis*, *Staphylococcus aureus*	18	1	2	Control	No	No	Yes
3	8	Yes	Tonsillitis	N/A	9	1	3	Control	Yes	Yes	Yes
4	6	Yes	Common cold	N/A	≤ 8	1	2	Intervention	No	No	Yes
5	12	No	Common cold	N/A	9	1	1	Intervention	No	No	No
6	69	Yes	Tonsillitis	N/A	34	1	3	Intervention	Yes	Yes	Yes
7	9	Yes	Pharyngitis	Metapneumovirus, coronavirus, *M. catarrhalis*	≤8	1	2	Control	No	No	Yes
8	10	Yes	Pharyngitis	None	39	1	3	Control	Yes	Yes	Yes
9	7	Yes	Tonsillitis	N/A	71	3	5	Intervention	Yes	Yes	Yes
10	7	No	Common cold	GAS, *Hemophilus influenzae, Streptococcus pneumoniae*	90	1	1	Control	No	Yes	Yes
11	22	Yes	Pharyngitis	None	120	1	2	Control	No	No	Yes

CRP = C-reactive protein; GAS = group A *Streptococcus*; N/A = not applicable, test not performed, NP tested with TAC.

* Centor score: limited to a maximum of three because no cervical lymph node examinations were recorded.

‡ Antibiotic taken at days 0–14 and includes those prescribed during the study and any sourced from elsewhere.

‡ Symptoms resolved by day 14, as reported by the patients.

Only four (36.4%) were prescribed an antibiotic at the index consultation, and one additional patient in the control group with high CRP was prescribed an antibiotic on the return visit at day 5. None of the patients sourced antibiotics from elsewhere during the 2 weeks of follow-up. Patients reported symptom resolution by day 14 in 10/11 (90.9%) cases. One patient had a persisting cough, but their other symptoms (fever, sore throat, and runny nose) had resolved.

Median CRP values for each swab result are shown in Table 4; there were two missing CRP values at enrollment into the main RCT. The median CRP levels were signficantly higher in patients with GAS than in those with no BHS isolated (*P* = 0.0302), and higher in those with any BHS isolated (*P* = 0.0516). C-reactive protein > 8 mg/L had a sensitivity of 81.8% (95% CI 52.3–94.8%) and specificity of 47.4% (95% CI 39.1–55.8%) for the detection of GAS compared with no BHS isolation. There were no significant statistical relationships between CRP values > 8 mg/L (*P* = 0.112), Centor scores ≥ 3 (*P* = 0.212), and FeverPAIN scores ≥ 4 (*P* = 1.000) and diagnosis of GAS compared with no BHS isolation.

**Table 4:** Median CRP values by throat swab results

	Number of patients, *n*/*N* (%)	CRP Median (IQR)	CRP ≤ 8 mg/L, *n*/*N* (%)	*P*-value*
No sore throat†	618/1,182 (52.3)	≤ 8 (≤ 8–13)	382/617 (61.9)	N/A
Sore throat‡	564/1,182 (47.7)	9 (≤ 8–19)	280/563 (49.7)	N/A
Sore throat and BHS not isolated	134/169 (79.3)	10 (≤ 8–18)	63/133 (47.4)	–
Sore throat and any BHS positive	35/169 (20.7)	14 (≤ 8–38)	12/35 (34.3)	0.0516
Sore throat and group A *Streptococcus*	11/169 (6.5)	18 (9–71)	2/11 (18.2)	0.0302
Sore throat and group C or G streptococci	18/169 (10.8)	11 (≤ 8–26)	8/18 (44.4)	0.5663
Sore throat and other BHS	6/169 (3.6)	17.5 (≤ 8–38)	2/6 (33.3)	0.2439

BHS = β-hemolytic *streptococcus*; CRP = C-reactive protein; N/A = not applicable.

* Ranksum test to compare CRP values of positive throat swabs against no BHS isolation.

† Presenting with an acute fever or history of fever to the main RCT.

‡ Presenting with an acute fever or history of fever and a sore throat to the main RCT.

## DISCUSSION

This study provides an estimate of GAS prevalence in those presenting with a sore throat to primary care units in northern Thailand. It confirms that GAS isolates remain fully sensitive to penicillin but show moderate levels of resistance to erythromycin, clindamycin, and chloramphenicol. The agreement between CRP, Centor, and FeverPAIN clinical scores and the detection of GAS isolates was poor.

### Group A *Streptococcus* prevalence.

Group A *Streptococcus* was found in 6.5% of patients presenting with a sore throat and history of fever. This is lower than estimates from a meta-analysis of more than 495,000 patients presenting to primary care or emergency departments, where 24.1% of those presenting with symptoms of pharyngitis or URTI were found to have GAS on culture.^29^ They found higher prevalence in high-income countries than LMICs, at 24.3% and 17.6%, respectively.^30^ Our findings were similar to other studies from Thailand, which found GAS in 3.8–7.9% of patients presenting with URTI,^3,31^ and a study of influenza-like illnesses in Lao PDR (8.6%).^32^

### Antibiotic susceptibility.

Reported antibiotic resistance levels vary across the globe and over time. In our study, we found that all of our isolates were sensitive to penicillin and ceftriaxone, despite reports of ceftriaxone resistance emerging from Iran, which needs further exploration and confirmation, as historically GAS has been fully susceptible to penicillins and cephalosporins.^33–35^ Erythromycin resistance was found in 2/11 (18.2%) GAS isolates, an important antibiotic for the treatment of pharyngitis in those with penicillin allergies. The national AMR surveillance center reported lower levels (7.2%) of erythromycin resistance in isolates from 85 hospitals across Thailand in 2018.^36^ The resistance levels in our study are lower than those reported from India (46%),^37^ Iran (33.9%),^33^ and South Korea (25.7%),^38^ similar to those from Greece (18.8%)^12^ but higher than those from the United States (6.8%).^13^ Reports of macrolide resistance are causing concern and limiting their use as empirical treatment for sore throats.^12,13,35^ Cross-resistance between macrolides and lincosamides (e.g., clindamycin) can occur because of *erm* genes.^34^ Clindamycin and erythromycin resistance was found in 2/11 (18.2%) of our isolates; this is higher than reported clindamycin resistance in the United States (0.5%),^13^ Greece (1.4%),^12^ the Thai national data (6.4%),^36^ India (9.5%),^37^ Iran (13.5%),^33^ and South Korea (15.8%).^38^ Resistance levels of 18.2% to chloramphenicol are much higher than the 0.8% reported in Greece^12^ and the 7.1% in Thai national data.^34^

These results from our limited number of GAS isolates indicate that narrow-spectrum penicillin should remain the mainstay of treatment for GAS pharyngitis in Thailand, as elsewhere.^11,39^ It is thus a concern that none of the 53 patients with sore throat and fever who received antibiotics had received penicillin (almost all received amoxicillin). This finding is not limited to this study but was also seen in all primary care units in the same district of Chiang Rai in a previous review of antibiotic use in primary care and is consistent with the Thai antibiotic guidelines, which give the option of treatment with penicillin V or amoxicillin for GAS pharyngitis.^4,5^ Given the current concern about the development of AMR, largely fueled by overuse of antibiotics,^40,41^ where possible it is intuitive to use narrow-spectrum (i.e., penicillin V) rather than broad-spectrum (i.e., amoxicillin) antibiotics. Caution is also required with the use of erythromycin, and surveillance of AMR should be conducted and used to update treatment guidelines.

### Who should receive antibiotic prescriptions?

Diagnosing GAS and deciding whether antibiotics are needed remain challenging, and the best approaches will vary depending on the setting, availability of health care and diagnostics, as well as the prevalence of GAS infections, carriage, and complications such as rheumatic heart disease.^7,21,42^ In Thailand, where a comprehensive primary healthcare system exists, most of the population have access to universal health coverage, and antibiotics are available from pharmacies and stores.^43,44^ Diagnostic tests for GAS are not routinely available in primary care. The prevalence of GAS is relatively low (6.5% in our study), and rheumatic heart disease is uncommon.

In our cohort, 53/169 (31.4%) patients were prescribed antibiotics, and despite only a third of GAS cases being prescribed antibiotics at the index consultation, all but one reported symptom resolution by day 14. Depending on the guidance followed, prescription rates would vary greatly within this cohort of patients. Using isolation of GAS from a throat swab as the indicator for antibiotic treatment in accordance with current guidance, 11/169 (6.5%) cases would require antibiotics, and this would increase to 29/169 (17.2%) if group C and G *Streptococci* were also treated. If a CRP threshold of more than 8 mg/L had been used, 93/168 (55.4%) patients would have been prescribed an antibiotic, missing 2/11 (18.2%) patients with the presence of GAS. This may seem like a low CRP cutoff clinically, but the medium CRP level for those presenting with a sore throat and fever was 9 mg/L. In primary care, diagnostic tests are mostly used to rule out rather than rule in serious infections, and increasing the CRP cutoff would reduce its sensitivity. With a Centor score of 3 or 4, 4/169 (2.4%) patients would require an antibiotic compared with 18/169 (10.7%) patients with a FeverPAIN score of 4 or 5, with a further 120 (71.0%) potentially being prescribed antibiotics because of a FeverPAIN score of 2 or 3. All of these interventions would reduce antibiotic prescription compared with routine care, where 88.4% of patients diagnosed with tonsillitis or pharyngitis in this setting are prescribed an antibiotic (almost exclusively amoxicillin).^4^

Of these strategies, use of throat swabs for all patients is impractical in the Thai primary care setting. Clinical scores are feasible, although whether they reliably identify GAS infection is questionable as indicated by our data and previous studies.^25,42^ Current high prescription rates in routine care also indicate that healthcare workers have limited confidence in withholding antibiotics based on clinical scores alone.^4^ Point-of-care CRP tests give results within minutes, making their use in primary care a feasible option. However, although patients with GAS had significantly higher median CRP levels, the association between a CRP > 8 mg/L, Centor scores of ≥ 3, and FeverPAIN scores of ≥ 4 and GAS detection was poor. Withholding antibiotics from patients with a CRP of ≤ 8 mg/L would miss 2/11 (18.2%) patients with positive throat swabs for GAS. This could be explained by the imperfect sensitivity of CRP but could also be due to GAS carriage rather than active infection. Both patients had a Centor score of 1 and FeverPAIN score of 2; neither had antibiotics prescribed at the index consultation, yet both had symptom resolution by day 14. The specificity of CRP testing to identify pharyngitis caused by GAS is poor as CRP can be raised in other infections and inflammatory processes.^45^

### Limitations.

The major limitations of our study are the small numbers of GAS isolates and the retrospective calculation of the Centor and FeverPAIN clinical scores. Cervical lymph node examination was not recorded, limiting the possible Centor score to 3 rather than 4. This could lead to the underestimation of Centor scores and may have affected the ability of the scores to identify pharyngitis caused by GAS. The FeverPAIN scores may also be imprecise as the presence of tonsillar exudate and inflamed tonsils were collected from descriptions of abnormal throat examination. However, we may have overestimated the number of patients with fever in the last 24 hours as 73.4% of patients had a history of fever lasting more than one day.

Another limitation is that we do not have throat swabs from healthy control patients to determine the carriage levels of GAS in the general population. A meta-analysis from 2018 estimated 7% GAS carriage levels in all ages in high- and low-income settings (8.0% in children and 2.8% in adults).^30^ It is also possible that throat swabs and culture may not have identified all GAS cases. This study was nested within a larger clinical trial on point-of-care CRP testing to guide antibiotic use in febrile illnesses. Finally, the rate of antibiotic prescribing in this study was relatively low and may have been influenced by the observer effect.

Given the limitations of our study, further validation of these indicators for antibiotic prescription for GAS pharyngitis in Thailand is required. A combination of clinical scores and CRP testing may offer the most practical and effective approach.

In conclusion, less than 10% of patients presenting to primary care in Thailand with a sore throat and history of fever had GAS detected. Penicillin is the most appropriate empirical antibiotic for those needing treatment. Surveillance of macrolide resistance is required, and susceptibility testing may be warranted for those with penicillin allergies. Identifying which patients with pharyngitis require antibiotic prescription remains challenging and requires further data from larger studies. However, despite our small sample size, CRP levels were higher in patients from whom GAS was isolated than in those without BHS isolation. Relying on CRP testing alone would result in high levels of antibiotic prescription for sore throats (49.7%), but in some settings, including Chiang Rai, this would still represent an important reduction in antibiotic prescription compared with routine care. Targeted CRP testing through the use of clinical scores is likely to be the most cost-effective approach to ruling out GAS infection.

## Supplemental materials

Supplemental materials
