# Supplementary material for: Prevalence of Group A Streptococcus in Primary Care Patients and the Utility of C-Reactive Protein and Clinical Scores for Its Identification in Thailand
Source: Am J Trop Med Hyg. 2019 Dec 30;102(2):377–83. doi: 10.4269/ajtmh.19-0502 (PMC7008346; doi:10.4269/ajtmh.19-0502)
Supplement: Supplementary file 1 [file tpmd190502.SD1.pdf]

Supplementary Material

NP swabs from the control group were tested using TaqMan® Array Card (TAC) with ViiA 7 real-time PCR system (machine and cards produced by Thermo Fisher Scientific, Waltham, MA), for the following 15 bacterial, 16 viral and 1 fungal pathogens; *Acinetobacter baumannii, Bordetella pertussis, Bordetella parapertussis, Burkholderia pseudomallei, Corynebacterium diphtheriae, Chlamydia pneumoniae, Chlamydia trachomatis,* Group A *Streptococcus, Haemophilus influenzae, Klebsiella pneumoniae, Moraxella catarrhalis, Mycoplasma pneumoniae, Pseudomonas aeruginosa, Staphylococcus aureus, Streptococcus pneumoniae,* adenovirus, human bocavirus, cytomegalovirus, common human coronavirus, Middle East Respiratory Syndrome coronavirus (MERS-CoV) , enterovirus, hepatitis E virus, influenza A, influenza B, human metapneumovirus, measles virus, parainfluenza, rhinovirus, respiratory syncytial virus, rubella virus, varicella zoster virus and *Pneumocystis jirovecii.*
